# Characterizing Spatiotemporal Dynamics of Methane Emissions from Rice Paddies in Northeast China from 1990 to 2010

**DOI:** 10.1371/journal.pone.0029156

**Published:** 2012-01-03

**Authors:** Yuan Zhang, Shiliang Su, Feng Zhang, Runhe Shi, Wei Gao

**Affiliations:** 1 Key Laboratory of Geographical Information Science, Ministry of Education, East China Normal University, Shanghai, China; 2 Research Center of Remote Sensing & Geoscience, Northeast Institute of Geography & Agroecology, Chinese Academy of Sciences, Changchun, China; 3 College of Environmental & Resource Sciences, Zhejiang University, Hangzhou, China; 4 Key Laboratory of Arid and Grassland Agro-Ecology, Lanzhou University, Lanzhou, China; University of Western Australia, Australia

## Abstract

**Background:**

Rice paddies have been identified as major methane (CH_4_) source induced by human activities. As a major rice production region in Northern China, the rice paddies in the Three-Rivers Plain (TRP) have experienced large changes in spatial distribution over the recent 20 years (from 1990 to 2010). Consequently, accurate estimation and characterization of spatiotemporal patterns of CH_4_ emissions from rice paddies has become an pressing issue for assessing the environmental impacts of agroecosystems, and further making GHG mitigation strategies at regional or global levels.

**Methodology/Principal Findings:**

Integrating remote sensing mapping with a process-based biogeochemistry model, Denitrification and Decomposition (DNDC), was utilized to quantify the regional CH_4_ emissions from the entire rice paddies in study region. Based on site validation and sensitivity tests, geographic information system (GIS) databases with the spatially differentiated input information were constructed to drive DNDC upscaling for its regional simulations. Results showed that (1) The large change in total methane emission that occurred in 2000 and 2010 compared to 1990 is distributed to the explosive growth in amounts of rice planted; (2) the spatial variations in CH_4_ fluxes in this study are mainly attributed to the most sensitive factor soil properties, i.e., soil clay fraction and soil organic carbon (SOC) content, and (3) the warming climate could enhance CH_4_ emission in the cool paddies.

**Conclusions/Significance:**

The study concluded that the introduction of remote sensing analysis into the DNDC upscaling has a great capability in timely quantifying the methane emissions from cool paddies with fast land use and cover changes. And also, it confirmed that the northern wetland agroecosystems made great contributions to global greenhouse gas inventory.

## Introduction

Methane (CH_4_) is a major greenhouse gases (GHG). According to the Intergovernmental Panel on Climate Change (IPCC) report, atmospheric CH_4_ concentrations have risen to 1774 ppb in 2005 [1]. Many studies have proved that agricultural activities are responsible for approximately 50% of global atmospheric inputs of CH_4_, wherein the rice paddies have been identified as a major source [2]. Over 10% of atmospheric CH_4_ was attributed to the emissions from global rice paddies [3], [4]. Thus, how to accurately estimate the CH_4_ emissions from rice paddies has become an pressing issue for assessing the environment impacts of agroecosystems, and further making GHG mitigation strategies at regional or global levels.

As an important rice producing country, China possesses approximately 20% of the world's rice paddies which provides about 30% of the world's rice needs [5]. About 20% of all croplands in China were cultivated for rice production [6]. Such a huge CH_4_ source could make a great contribution to global CH_4_ inventory. To estimate the national inventory of CH_4_ emission, a number of site-specific observations were conducted for measuring CH_4_ flux at field sites in the major rice producing areas in Southern and Southeast of China [7]–[10]. These ground-based measurements were reliable for understanding the mechanics of CH_4_ emission at local scale. Given the emergence of new frameworks for GHGs mitigation, however, it fails to respond to practicable requirements at national, regional, and global levels in the long run for high variations in spatial and temporal pattern of CH_4_ emission with changing environmental conditions [11]–[15]. Consequently, based on the extrapolation of the understandings gained at site scale to a large spatial dimension, model simulations were required to meet the demands for spatiotemporal analysis of CH_4_ emissions from rice fields.

Model estimation of CH_4_ emissions from rice fields began with empirical models based on the regression relationships between CH_4_ emission rate and rice biomass or yield [16]–[18]. However, these “easy-to-use” approaches were unable to reasonably explain biogeochemical processes involved in CH_4_ production, oxidation and emission, and also explicitly delineate the emissions variation at regional scale across a wide range of soil conditions and management practices. In this case, many physical models consequently were developed based on biogeochemical process to quantify the comprehensive effects of ecological drivers, soil and climate factors and management alternatives on agricultural production and environment [19]–[21]. For their capability in simulating CH_4_ production and oxidation process in paddies, so were extensively utilized to regional or global CH_4_ estimation [22]–[27].

Of among, the Denitrification and Decomposition (DNDC) model is a generic model that simulates the biogeochemical processes leading to GHG emissions from soil [19], [28], [29]. It has been adapted to simulations of GHG (e.g. CO_2_, N_2_O or CH_4_) emissions from a wide range of systems such like dryland crop, pasture, rice paddy, and forest systems [30]. For rice-cropping system, DNDC has been substantially tested/validated against observed CO_2_, N_2_O or CH_4_ fluxes during the past two decades [25], [31]–[33]. Satisfactory results were achieved in a number of countries across the world like the U.S., China, Thailand, India, Japan, etc. [32]–[36]. Many studies proved that DNDC is most applicable for estimating CH_4_ emissions from rice paddies at regional scale [26], [27].

To implement the upscaling for multi-temporal, regional CH_4_ estimation, accurate acquisition of spatial distribution of rice field was indispensable to advance the regional applications of DNDC. Remote sensing (RS) for mapping rice could provide more accurate spatial information of rice fields than conventional census data. Many researchers utilized remotely sensed data (optical or microwave) for mapping the spatial distribution of paddy rice at regional scale [37], [38]. DNDC has been discussed for upsacling by integrating the RS technique to compile greenhouse gas inventories, identify spatial patterns in emission, or explore scenarios for GHG mitigation [39]–[41].

In this study, a representative region of paddy rice production, the Three-Rivers Plain (TRP) in Northern China, was selected for regional CH_4_ estimation. This region possesses climate, soil and management conditions differing from that in the tropical or subtropical rice regions. With the introduction of RS analysis into the DNDC upscaling, this study aims at characterizing the spatiotemporal patterns of the CH_4_ emissions from rice fields in the TRP over the past two decades (from 1990 to 2010), and further for quantifying the contribution of the rice paddies within the TRP to global methane.

## Methods

### Study area

**Figure 1:**
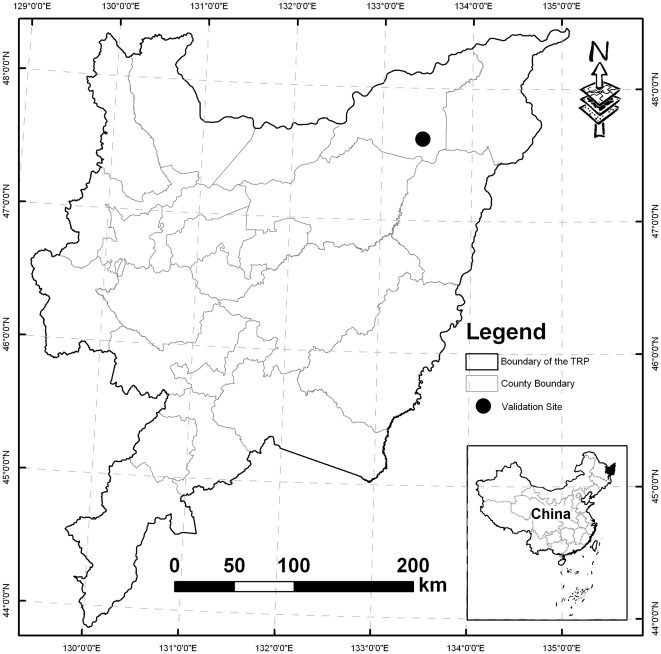
Location of the Three-Rivers Plain.

The study area, the TRP, is located in northeast China (48.5°–43.8°N and 129.2°–135.1°E). Three major rivers, Songhua River, Wusuli River and Heilong River, whose watersheds cover almost the entire territory (10.93 million hectares) of the eastern part of Heilongjiang Province (Fig. 1). This region lies at 45 to 60 m geographic elevation above sea level with a gentle and flat topographic relief. Croplands is cultivated in this region from early May to early October for each cropping year with leaving fields fallow for a long period (approximately 7 months). Annual mean temperature is ranged from 2.6 to 5.2°C, and annual precipitation ranged from 330 to 850 mm during the period of 1980–2010. The soils are fertile and rich in organic matter. The flat topography, fertile soils and abundant water resources have made the alluvial plain favorable for crop production. In the 1950s, land reclamation campaigns in the Northeast China converted a majority of the natural swamplands into farmlands. Over the past six decades, the region has experienced drastic changes in the land use. Especially in the recent 20 year, over one million ha of lands has been cultivated as rice paddy.

The rice fields in the TRP, with the highest latitude in not only China but also the world, are one of the most important resources producing high quality rice for the region even the whole county. In this region, a small population of farmer owes a large amount of agro-lands, and modern cultivation managements are extensively practiced. Only single-season rice is planted in the region with growing season from late May to late September. Continuously deepwater flooding (with >10 cm water depth) is widely adapted in rice fields cross the TRP. Urea and synthetic fertilizer are predominantly applied without any organic matter amended. About 10% of the rice straw is normally left as stubble in the fields after harvest in October, and the stubble is incorporated into the soils with tillage before the beginning of the next rice season.

### Model Validation

To validate the applicability of DNDC model for the rice fields in the TRP, field experiments were conducted at a paddy site in the Honghe Farm (at 47°35′N and 133°31′E) in 2004 and 2006 within the plain (Fig. 1). Ecological factors to drive DNDC model for simulating the CH_4_ production and oxidation in rice paddy included three major factors, i.e. climate, soil properties and management practices. These factors (i.e. model inputs) were used to run DNDC for the experimental site. Daily meteorological data (air temperature and precipitation) were acquired from the local climate station, a part of the Ecological Experimental Station of Mire-Wetland in the TRP run by the Chinese Academy of Science. Soil physical and chemical properties in the field site were obtained from the ground-based measurements. Rice cultivation information was collected from the log documents of field measurements. Three treatments were conducted in a same paddy field with specific management practices (see details in [41]). Three treatments are: 60 kg N/ha of N-fertilizer application rate in 2004 (T1); 150 kg N/ha of N-fertilizer application rate in 2004 (T2); and 150 kg N/ha of N-fertilizer application rate in 2006 (T3). The measurements of CH_4_ fluxes were conducted twice per week with static chamber method through the rice-growing period (from late May to early October). The measured CH_4_ flux data were used to compare with the modeled CH_4_ fluxes at daily time step. Statistical tools such as the root mean square error (RMSE), the coefficient of model efficiency (EF) and the coefficient of model determination (CD) were adopted to assess the “goodness of fit” of model predictions. Normally, value for EF is less than or equal to 1. A positive value indicates that the simulated values describe the trend in the measured data better than the mean of the observed values. The CD value is larger than or equal to 0. When a CD value of 1 or above, it indicates that the model describes the measured data better than the mean of the observations. Taken together, EF and CD allow RMSE to be further interpreted where standard error values of the measurements are unavailable. Detailed description on the calculation of the RMSE, CD and CD were listed in [42].

### Construction of database for Regional Simulation

For the purpose of characterizing the spatiotemporal patterns of CH_4_ emissions, it is necessary to construct geographic information system (GIS) database for regional simulation with DNDC upscaling. This database included multi-temporal rice field maps, soil properties, daily weather data, and farming management practices cover the study region.

To delineate the spatial distribution of rice fields in the TRP, three rice maps were retrieved from Landsat thematic mapper (TM) RS imagery acquired in approximately 1990, 2000 and 2010 (Table 1). Total of 30 TM images with a high spatial resolution of 30 m were selected in the tillering or near-mature stage of rice in 1990 (9 images), 2000 (9 images) and 2010 (12 images). These Landsat TM images accessed from the EarthExplorer Interface (http://edcsns17.cr.usgs.gov/EarthExplorer/) were digitized by visual interpretation technology at the GIS software environment of ArcGIS 9.2, which were used to extract the detailed spatial distribution of paddies in the TRP. In terms of the unique phenology features of rice, three accurate rice maps were successfully extracted from the clear remote sensing images.

**Table 1:** Remotely sensed Landsat TM imagery used for retrieving rice paddy.

	Acquisition date		
Path/Row No.	1990	2000	2010
113/26	-	-	19/09/2010
113/27	19/10/1992	05/09/2002	19/09/2010
113/28	-	-	19/09/2010
113/29	-	-	19/09/2010
114/27	12/06/1989	11/08/2002	06/06/2010
114/28	16/09/1989	25/09/2001	06/06/2010
114/29	29/09/1988	25/09/2001	-
115/27	25/06/1991	12/08/2000	14/09/2009, 17/09/2010
115/28	02/09/1993	12/08/2000	14/09/2009
115/29	17/10/1992	31/08/2001	14/09/2009
116/27	04/09/1991	07/09/2001	08/09/2010
116/28	23/05/1994	07/09/2001	08/00/2010

The climate dataset were composed of daily maximum and minimum air temperature, precipitation and mean wind speed observed in 1990, 2000 and 2010. They were acquired from 7 basic weather stations in China (China Meteorological Data Sharing Service System at http://data.cma.gov.cn/). The entire plain was segmented into 7 sub-regions at which one weather station was located. We assumed the rice growth was with the same climate condition within each sub-region.

Soil data were derived from the soil dataset developed by the Institute of Soil Science, Chinese Academy of Sciences, which was compiled based on the second national soil survey of China conducted in 1980–1990s [43], [44]. The soil spatial dataset was a grid data with cell size of 10 km×10 km, which contains soil texture (sand, silt and clay percentage) and physical and chemical properties (e.g. organic matter, pH and bulk density) at multiple-layer profile. In the TRP, there were 605, 815 and 826 soil cells cover the rice paddies of the TRP in 1990, 2000 and 2010, respectively. Each cell containing soil properties in the top layer (0–10 cm) were used to drive the DNDC simulations. And then, spatial overlay analysis was performed to segment three rice thematic maps with the soil data with cell size of 10 km×10 km.

Generally, unlike the extensive farmland use mode in South China, the management practices are relatively identical cross the study region. The indistinctive differences in rice cultivation practices make less variation in CH_4_ emissions from rice paddy in this study area. Therefore for the multiple temporal simulation of CH_4_ emission, we assumed the general management practices in each simulated year was spatially identical for the entire rice paddies in TRP. Detailed information on paddy cultivation (e.g. planting/harvesting date, tillage/irrigation regime, fertilizer application, residue management, etc.) in 1990, 2000 and 2010 were investigated by communicating with a number of local agronomists and farmers (Table 2).

**Table 2:** Management practices on the rice paddies in the TRP.

Items	1990	2000	2010
Tillage	5/15: Plow depth of 20 cm	5/15: Plow depth of 20 cm	5/14: Plow depth of 20 cm
Rice cultivation	5/25: transplanting; 9/25: harvesting, grain yield of 2400 kg C/ha	5/25: transplanting; 9/25: harvesting, grain yield of 2700 kg C/ha	5/25: transplanting; 9/25: harvesting, grain yield of 3000 kg C/ha
Flooding	5/15–8/25: Continuously flooding, water depth of 10 cm	5/15–8/25: Continuously flooding, water depth of 10 cm	5/14–8/25: Continuously flooding, water depth of 10 cm
Fertilization	6/1: Urea (24 kg N/ha); 7/1: Urea (36 kg N/ha)	6/1: Urea+Synthetic fertilizer (36 kg N/ha); 7/1: Urea (54 kg N/ha)	1/1: Urea+Synthetic fertilizer (48 kg N/ha); 7/1: Urea (72 kg N/ha)
Manure application	No	No	No
Residues incorporation	10%	10%	10%

The spatially differentiated information above listed was compiled in the GIS database of DNDC for the regional simulation. DNDC was performed for characterizing the spatiotemporal patterns of CH_4_ emission with three input datasets in 1990, 2000 and 2010, respectively. DNDC run twice for each grid cell with the maximum and minimum values of the soil properties, which formed a range of CH_4_ emission that was later used for quantifying the uncertainty generated from the DNDC upscaling [25]. Based on the modeled CH_4_ flux and the rice field acreage within each cell, the total yearly CH_4_ emissions from the cell could be calculated. Three cell-level spatial patterns of CH_4_ emissions were mapped for the whole plain, and then the regional emissions were accumulated for evaluating their contributions to global CH_4_ inventory.

## Results

### Validation results and sensitivity factors analysis

**Figure 2:**
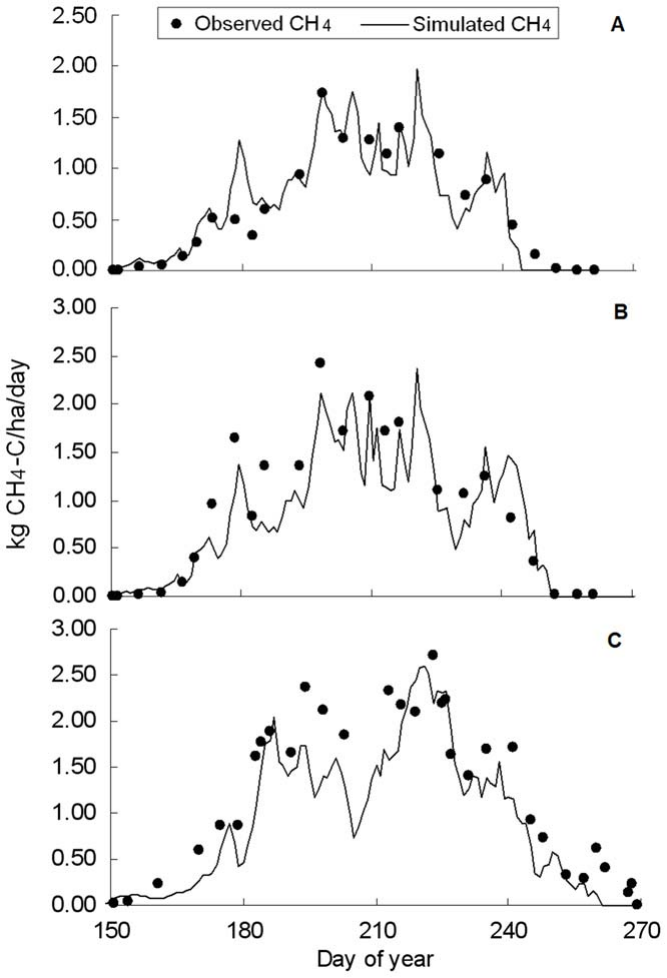
Simulated vs. observed CH_4_ fluxes in validation site. (A) 60 kg N/ha of N-fertilizer application rate in 2004, (B) 150 kg N/ha of N-fertilizer application rate in 2004, (C) 150 kg N/ha of N-fertilizer application rate in 2006. (data from Zhang et al., 2011).

The measured CH_4_ fluxes at the three treatments mentioned above were compared with modeled results. Figure 2 shows the comparisons between the modeled CH_4_ fluxes with observations. As a whole, the modeled results showed a fair agreement with observations although minor discrepancies exist across the three treatments. Results showed that the RMSE values were 0.190, 0.304 and 0.344 for treatment T1, T2 and T3, respectively. The EF values were positive (>0.8), and the CD values were greater than 1 for all the three treatments. Validation test has proved that the DNDC is capable of better capturing the seasonal behaviors of CH_4_ fluxes from the experimental site within the study area [41], [45].

**Figure 3:**
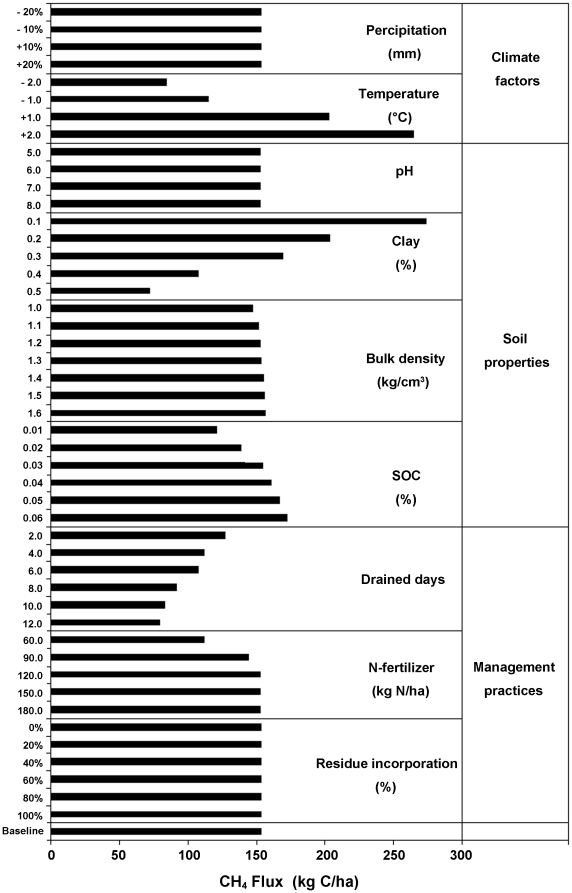
Sensitivity tests of environment factors driving CH_4_ emissions from rice paddies.

Methane production and oxidation in rice fields are controlled by many factors such as climate variables, soil properties, or agricultural management practices [46]. The sensitivity test provided crucial information for finding out the most sensitive factors from all input parameters, which could affect the modeled results for regional estimations. In the sensitivity test, baseline scenario was first set based on the average climate, soil and management conditions cover the study region. The simulated result from baseline scenario was taken as a benchmark of CH_4_ emissions for accessing those of other scenarios. And then, within a predefined range, DNDC were performed by varying single one of all input parameters while keeping all other input parameters constant (Table 3). The model responses to changes of these factors on CH_4_ emission from rice paddies in the TRP were presented respectively in Fig. 3.

**Table 3:** Environmental Factors for sensitivity tests.

Environmental Factors		Baseline value	Range tested
Climate	Annual mean temperature (°C)	3.8	1.8–5.8
	Total annual precipitation (mm)	550	440–660
Soil Property	Clay fraction (%)	0.32	0.1–0.6
	Initial soil C fraction (%)	2.78	1.0–6.0
	Bulk density (g/cm^3^)	1.25	1.0–1.6
	Soil pH	6.6	5.0–8.0
Management Practices	Depth of continuous flooding water (cm)	10	Not varied
	Drained days (d)	0	2–12
	Residue incorporation (%)	10	0–100
	N-Fertilizer application (kg N/ha)	150	60.0–180.0

Specifically, the response of CH_4_ emission to changes in climate factors was investigated by running DNDC using alternative climate scenarios. The modeled results indicated that precipitation changes (±20%) have no significant impacts on CH_4_ emissions for the continuously flooded paddies, whereas the fluctuation of CH_4_ emissions incurred by temperature changes (±2°C) was remarkable. The higher is temperature, the more CH_4_ emitted from rice paddy (Fig. 3). Temperature is therefore the most sensitive climate factor. This could be explained that the higher temperature accelerated the decomposition and fermentation process of soil organic matter (SOM) [47].

The spatial heterogeneity of soil property has a great impact on the pattern of CH_4_ emission. In this test, four major soil parameters (soil organic carbon (SOC) content, clay fraction, pH and bulk density) were tested within specific changing range (Fig. 3). Test results indicated that soil clay content was the most sensitive factor, followed by the SOC content. For the anaerobic soil flooded by deepwater, the sandy loam soil was more likely to produce more CH_4_ than the clay loam soil because large porosity existed in sandy soil could promote CH_4_ transmission and release from flooded fields. The soil with high SOC content could provide more dissolved organic carbon to the methanogens, which is favorable to CH_4_ production [25], [48]–[50]. In contrast, CH_4_ emission was less sensitive to both soil pH and bulk density.

Various scenarios on CH_4_ emissions with three major management practices (flooding regime, residue incorporation and N-fertilizer application rate) were simulated by DNDC (Fig. 3). There was no significant impact of the incorporation rate of rice straw and residue on CH_4_ emission from the incorporated fields. In the TRP, rice straw and residue were normally left in fields after rice harvest, no CH_4_ emitted from the fields which have been already drained over one month. The nitrogen level meeting the need of the optimal rice-grain production is 120 kg N/ha. CH_4_ emission increased with increasing N-fertilizer application when the applied amplitude was less than the optimal nitrogen demands for physical development of rice plant. When the application rate reached a level meeting the need for the optimal productivity, additional fertilizer application didn't make any more impact on CH_4_ emission. Flooded rice paddy provided favorable environment for methanogenesis. Mid-season drainage changed the anaerobic status of CH_4_ production, and thus reduced the CH_4_ emission from rice fields [51]–[53]. Simulated results showed that for the rice paddy in cool climate region the CH_4_ emission was reduced by 45% for 10 days draining duration in the peak tillering and early maturity stages. In the perspective of mitigating CH_4_ emission from rice paddy fields, mid-season drainage would become a potential opportunity through adjusting present management practices.

### Changes in rice paddies of the Three-Rivers Plain

**Figure 4:**
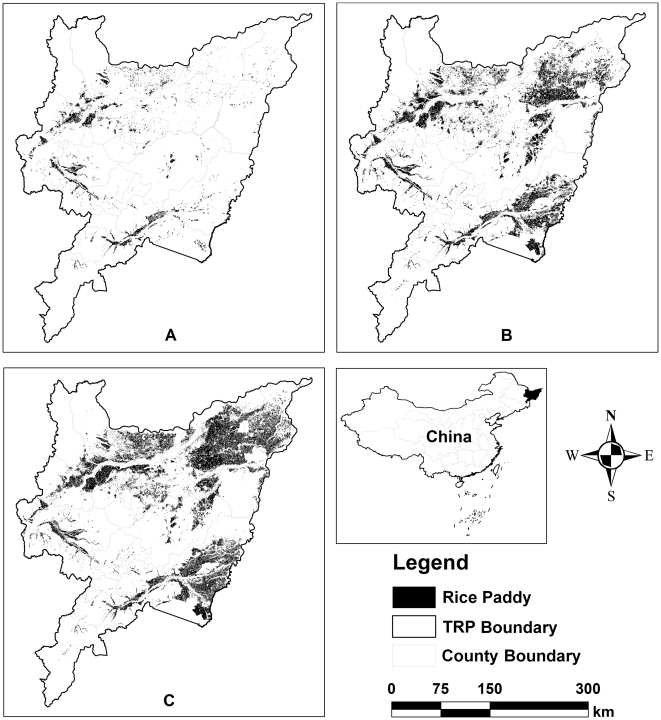
Rice paddies maps in the Three-Rivers Plain. (A) 1990, (B) 2000, (C) 2010.

Three accurate rice paddies maps cover the Three-Rivers Plain were delineated for each decade from 1990 to 2010 from TM images acquired in nominal 1990, 2000 and 2010 (Fig. 4). The rice paddies were mainly distributed in the lowland areas along with the major rivers. The extracted total area of paddy fields was 0.23, 1.22 and 1.63 million ha in 1990, 2000 and 2010, respectively. Statistical results showed that the rice area has increased approximate one million ha for the first 10 years (from 1990 to 2000), whereas about 0.4 million ha for the last 10 years (from 2000 to 2010). Although the trend in changes of total area of rice paddies was constant within the TRP, the increment rate was slowing down gradually. The results indicated the change intensity of land use/covers in the last decade was evidently smaller than the first one, which could be attributed to the limited available lands (reclaimed or converted) for new rice cultivation. From the perspective of spatial distribution, a majority of rice was cultivated in the western and southern lowlands of the plain in 1990. Some small patches of rice fields were sparsely distributed in the eastern of the TRP where the complexity of natural wetlands and drylands existed (Fig. 4 (A)). In contrast, a large amount of drylands have been extensively cropped as rice paddy since 2000 (Fig. 4 (B and C)). At present, the northeast and southeast have become the major area of rice production for the Three-Rivers Plain. These areas have been playing a role in providing the high quality rice and ensuring food security for the Northern China, as well as the country.

### Estimation of CH_4_ emissions for the Three-Rivers Plain

**Figure 5:**
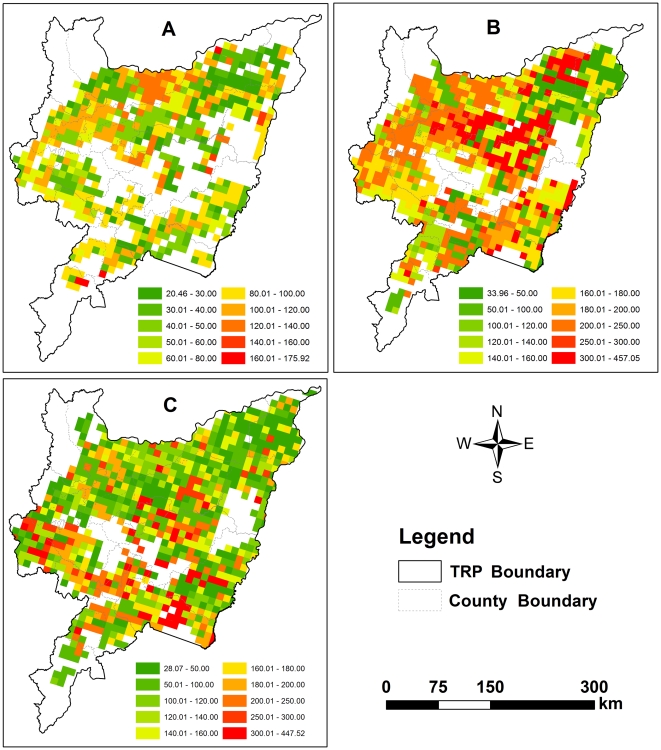
Mean CH_4_ emission rates (kg CH_4_-C/ha) of paddy fields. (A) 1990, (B) 2000, (C) 2010.

**Figure 6:**
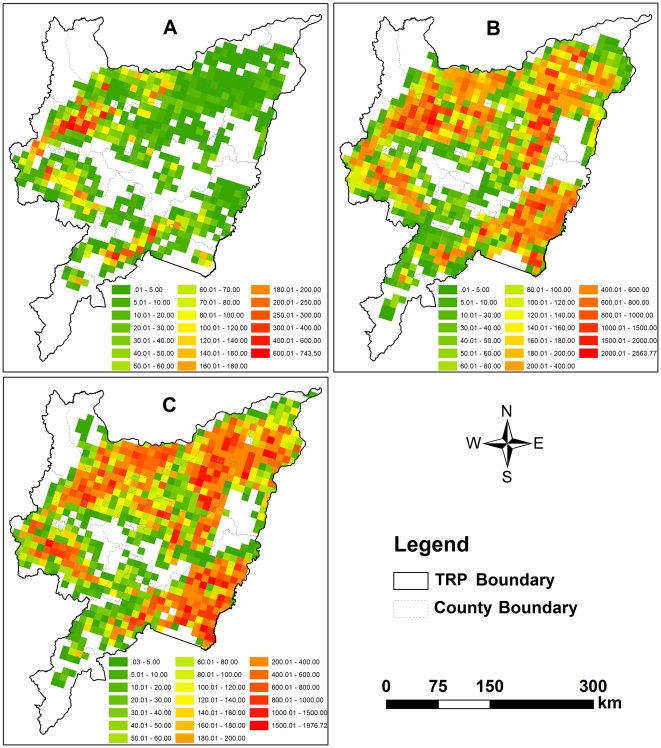
Yearly total CH_4_ emissions (ton CH_4_-C) from rice paddies. (A) 1990, (B) 2000, (C) 2010.

Driven by the three GIS database separately constructed for 1990, 2000 and 2010, DNDC simulated the CH_4_ flux cell-by-cell across the entire rice fields in the TRP. The total emissions were then calculated by multiplying the modeled flux by rice area in each cell to produce regional CH_4_ emissions for each simulated year. The spatial and temporal patterns of CH_4_ emissions were mapped cover the domain using GIS tools. Fig. 5 and Fig. 6 showed CH_4_ emission rates and total emissions at the cell scale, respectively. The two maps showed clear spatial patterns in CH_4_ emissions across the domain. Detailed descriptions were discussed as below.

In 1990, the simulated CH_4_ emission rates were 20.46–175.92 kg C/ha, and the average of 71.05 kg CH_4_-C were emitted from one hectare of rice field. Relatively higher CH_4_ flux (>120 kg CH_4_-C/ha) was located at a few of soil grid cells sparsely distributed in the southern tip and northern of the TRP, whereas lower flux (<30 kg CH_4_-C/ha) were in the most northeastern (Fig. 5 (A); Table 4). The western and southeastern of the plain, the major rice production area, emitted more CH_4_ (>200 tons (1 ton = 1000 kg)) than other areas. The northeastern was a lower emission area (<50 tons per cell) where a large number of farmlands were cropped as dryland crops at that time (Fig. 6 (A)).

**Table 4:** Simulated CH_4_ emission rates and total emissions for three simulated year.

Items		1990	2000	2010
Emission rates (kg C/ha)	Max.	175.9	457.05	447.52
	Min.	20.46	33.96	28.07
	Avg.	71.05	180.42	136.85
Total emissions per cell (ton C)	Max.	679.2	2563.77	1976.72
	Min.	0.01	0.01	0.03
	Avg.	36.44	258.85	245.54
	SD	73.01	348.92	329.09
Total emissions (Tg C)	Max.	0.027	0.228	0.241
	Min.	0.022	0.193	0.165
	Avg.	0.025	0.211	0.203

Significant changes in CH_4_ emission pattern happened in 2000. Higher CH_4_ flux (>250 kg CH_4_-C/ha) appeared in the central and the northern while the northeastern remained lower flux (<100 kg CH_4_-C/ha) (Fig. 5 (B)). The mean flux reach to 180 kg CH_4_-C/ha with large gradient of 34–457 kg CH_4_-C/ha (Table 4). Yearly total emission showed that those cells with high CH_4_ emission (>400 tons) accounted for over 40% of the domain (Fig. 6 (B)).

The recent CH_4_ emission pattern in 2010 was presented in Fig. 5 (C) and Fig. 6 (C). Those cell with higher CH_4_ flux (>250 kg CH_4_-C/ha) sparsely distributed over the entire rice paddies, and lower flux (<100 kg CH_4_-C/ha) in the northeastern kept constant. Although the simulated emission rates of CH_4_ in 2010 was slightly less than that in 2000 (Table 4), their patterns in total emission was very comparable (Fig. 6 (C)).

In summary, statistical results indicated that the regional average fluxes were around 71, 137 and 180 kg CH_4_-C/ha, in 1990, 2000 and 2010, respectively. The average of total CH_4_ emitted from the entire rice paddies of the TRP was 0.025, 0.211 and 0.203 Tg CH_4_-C (1 Tg = 10^9^ kg) in the three years (Table 4).

### Changes analysis for the spatiotemporal pattern in CH_4_ emission

From the simulated results of the TRP, the highest emission rate was 8–16 times higher than the lowest one in each of the three simulated years (Table 4). Such a huge difference in CH_4_ emission rate was due to the variations in the soil properties. Past study has demonstrated that those soil cell with lower CH_4_ flux contained higher clay fraction, and the higher flux normally occurred in those cells with relatively higher SOC content [41]. Consequently, the spatial patterns in CH_4_ fluxes in this study are mainly attributed to the Most Sensitive Factor (MSF) of soil properties, i.e. soil clay fraction and SOC content (Fig. 3).

As far as the yearly emissions from paddy cells were concerned, statistics analysis showed that the standard deviation (SD) of CH_4_ emission was 36.44 tons CH_4_-C in 1990, which was far less than that in 2000 and 2010 (258.85 and 245.54 tons CH_4_-C). Otherwise, although the minimum of yearly emission were very similar (0.01∼0.03 tons CH_4_-C) for all three simulated years, the maximum in 2000 and 2010 (>1900 tons CH_4_-C) were much more than that in 1990 (<680 tons CH_4_-C). Therefore, there was an observable emission variation cross the entire study region in 2000 and 2010 while the character in spatial variation was not distinct in 1990 (Fig. 4). Obviously the clear spatial variation in yearly CH_4_ emissions from cell data was attributed to the spatial distribution of rice fields.

In Table 4, average CH_4_ emissions (0.025 Tg) in 1990 was far less than in 2000 and 2010 (>0.2 Tg). The huge difference could be attributed to several reasons. First of all, total area of rice paddies was the primary determinant of total emission. Only 0.23 million ha of rice were planted in 1990. However since 2000, the rice paddy areas have exceeded 1.2 million ha (Fig. 4). Such rapid increase in rice planted area would inevitably lead to the consequential increase in total CH_4_ emissions. Additionally, the CH_4_ emissions rate in 1990 was also smaller than in other simulated years. This could be firstly related to the fact that more biomass production with more N-fertilizer application. The sensitivity tests have indicated that although the effects of N-fertilizer application rate on CH_4_ emissions seems not very evident, it did increase the crop biomass or yields that could indirectly enhance the CH_4_ production. In 1990s, only 60 kg N/ha of N-fertilizer rate were applied to the rice fields by compared with 90 and 120 kg N/ha of N-fertilizer rate in 2000 and 2010, separately.

**Figure 7:**
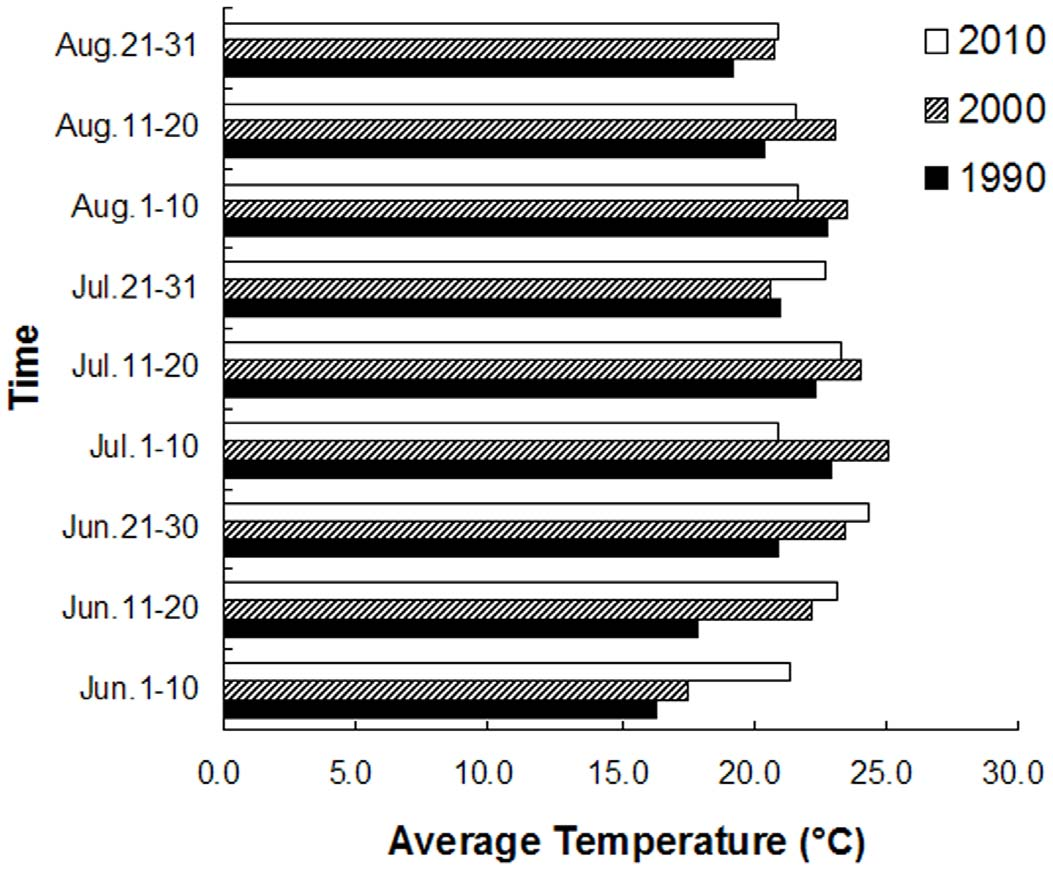
Average of 10-day temperature during the rice-growing season.

Otherwise, the temperature was a positive climate factor driving CH_4_ production from rice fields. The emission fluxes increased with temperature because the higher temperature accelerated soil organic matter (SOM) decomposition and fermentation process, which has been proved in past research [44], and also presented in the sensitivity test (Fig. 3). Although the rice area in 2010 was 30% more than that in 2000, total CH_4_ emissions (0.203 Tg CH_4_-C) in 2010 was slightly less than that (0.211 Tg CH_4_-C) in 2000. The results was largely related to this fact that higher flux (180 kg CH_4_-C/ha) in 2000 than that in 2010 (137 kg CH_4_-C/ha). By comparing the average of 10-day temperature from rice transplanting to harvesting stage (late May through September), the temperature during the rice-growing season in 2000 was apparently1.13–2.65°C higher than that in 2010 [Fig. 7]. Therefore, the temperature would be taken as a major factor for regional estimation of total CH_4_ emission from rice paddies at long term and large regional scale.

## Discussion

Rice paddy mapping with multi-temporal RS data can quantify the dynamics of cropland use. Since 1990, the agricultural lands have been experiencing significant changes in the TRP in the very northeastern part of China. In the major rice producing areas with the cooler climate in China, fast rise in rice cultivation was driven by the market demand for high yield/quality rice. More upland crops like corn or soybeans were converted to paddy rice in Northern China during past two decades. How to real-time estimate the GHG emission induced by this kind of rapid land-use transformation have been becoming an important objective for making the scientific GHG inventory for China. Consequently, characterizing and quantifying the spatiotemporal pattern of CH_4_ emissions from rice paddies could be an interested topic for researcher in the future.

In our precious study, DNDC upscaling has been utilized to quantify CH_4_ emissions from rice fields of the study area in 2006 after the elaborate calibration and site validation [41]. This present study extended previous study to multi-temporal CH_4_ estimation for characterizing spatial and temporal dynamics of CH_4_ emissions over past two decades (from 1990 to 2010). The modeled average of CH_4_ fluxes for the simulated year of 1990, 2000 and 2010 was 70, 180 and 137 kg CH_4_-C/ha, respectively. These results are comparative with the average emission rates observed and modeled in the Taihu Lake region of Southeast China (15–198 kg CH_4_-C/ha/year) [27].

In the past studies, a baseline emission factor of 1.30 kg CH_4_/ha/day in the 2006 IPCC Guidelines [54] was often recommended to estimate the regional or global CH_4_ emissions from rice paddies. Thus, With the prevail management practices of 100-day continuously flooded without organic amendments for rice paddies in the TRP, the total CH_4_ emissions based on the IPCC approach was 0.030, 0.159 and 0.212 Tg CH_4_-C in the 1990, 2000 and 2010, respectively. Comparative analysis indicated that the simulated values in 1990 and 2010 (0.025 and 0.203 Tg) were slightly less than that of IPCC estimation, whereas the simulated values (0.211 Tg) in 2000 were significantly larger than the IPCC estimation. In this study, we took into account the integrated influence of various ecological drivers to CH_4_ emissions from rice paddy. These drivers including climate, soil and management factor, were applied to drive DNDC model for regional CH_4_ estimation. Thus this method introduced in this study would make great improvement for the CH_4_ estimation compared to the IPCC method based on baseline emission factor.

Our modeled results further confirmed the high latitude wetland agroecosystems like rice paddy in Northern China was an important anthropogenic CH_4_ source. With the increase in the rice-growing area, the rice paddies in the TRP could make more contribution to global CH_4_ inventory. During the period of past decades in this region, natural swamp wetlands were first converted into dryland croplands and then, into anthropogenic wetlands (rice paddies). This kind of unique changes in land use/covers would consequentially resulted in huge environmental impacts. A number of ground-based observations on CH_4_ emissions from natural wetlands have been substantially conducted in the high latitude plain of China [55]. These studies provided important supports for regional estimation of CH_4_ emissions with spatial modeling technology. Thus quantifying the dynamics (i.e. the net increment) of CH_4_ emissions in the process of land transformation in this region would be an interested topic for researchers in the future.

During the recent years, China authority has paid more attention to GHG inventory and mitigation. Developing an effective method towards assessing the magnitude of impacts from rice-cropping systems would be significant for meeting the social and research needs. The results in this study demonstrated huge potential of integrating biogeochemical models with RS mapping technology for meeting the environmental challenges rise in coming years in China.
